# Congenital adrenal hyperplasia and vanishing testis: rare case of male pseudohermaphroditism

**Published:** 2016-03

**Authors:** Azam Ghanei, Golnaz Mohammadzade, Ehsan Zarepur, Sedigheh Soheilikhah

**Affiliations:** 1 *Department of Internal Medicine, Shahid Sadoughi University of Medical Sciences, Yazd, Iran.*; 2 *Student Research Committee, Shahid Sadoughi University of Medical Sciences, Yazd, Iran.*

**Keywords:** *Congenital adrenal hyperplasia*, *Vanishing testes*, *Ambiguous genitalia*

## Abstract

**Background:**

Congenital adrenal hyperplasia (CAH) and vanishing testes are uncommon diseases that can result from hormonal and mechanical factors. Classic CAH is determined by ambiguous genitalia and increase in amount of 17-Hydroxyprogesterone. Simultaneous occurrence of CAH and vanishing testes is a rare condition.

**Case:**

A 22-year-old boy, known case of CAH who was diagnosed as female pseudohermaphroditism due to ambiguous genitalia, was referred to Shahid Sadoughi Hospital, Yazd, Iran with colicky abdominal pain and hematuria. Ultrasonography has been performed and prostate tissue was reported. Karyotyping was done because of uncertainty in primary diagnosis, which revealed 46XY. For finding location of testes, ultrasonography and MRI were done and nothing was found in abdomen, inguinal canal or scrotum. Inhibin B serum level was measured to find out whether testis tissue was present in the body, which was <1 pg/ml and vanishing testis was confirmed.

**Conclusion:**

Early diagnosis and treatment are essential to prevent further sequels and karyotyping for all patients with CAH is recommended. Lifelong treatment with synthetic glucocorticoid replacement is necessary.

## Introduction

Congenital adrenal hyperplasia (CAH) refers to any of several autosomal recessive diseases resulting from deficiency of one of five enzymes required for synthesis of cortisol in adrenal cortex. Most frequent is 21-hydroxylase deficiency and about 1 in 16,000 children are born with classic 21-hydroxylase deficiency (1).

Classic 21-hydroxylase deficiency is characterized by elevated serum levels of 17-hydroxyprogesterone (1, 2). There is a wide spectrum of phenotypes. Clinical signs of classical 21-hydroxylase deficiency are observed prenatally or at birth and are subdivided into a salt-wasting form, which is more severe, and a simple virilizing form. non-classical type which is the least severe form, manifests later in life (3).

Testes function can lead to internal and external genital differentiation and development especially during fetal age. So, many factors can interfere this process. Congenital anorchia (vanishing testis syndrome) is a rare syndrome in which testes are absent in male person and is seen in˂5% of cryptorchidism cases. The specific factor has not yet been known. This disease can be unilateral or bilateral and commonly external genitalia are normal.

Diagnosis of vanishing testis syndrome is based on clinical presentation. Micropenis due to prenatal disorders is an important symptom in infants with bilateral anorchia. Also delayed puberty (pre pubertal primary hypogonadism) is a common complaint in males with congenital anorchia. On examination, physician cannot palpate testes and blind-ending spermatic cords are a common finding that shows the presence of the testis in early fetal life. Testosterone and gonadotropin concentrations are commonly low in this syndrome (4).

The aim of present report was to analyze the clinical and hormonal findings in a patient affected with congenital bilateral anorchia and CAH and also to determine any possible relationship with congenital adrenal hyperplasia.

## Case report

A 22-year-old boy, known case of CAH who was diagnosed as female pseudohermaphroditism due to ambiguous genitalia, was referred to Shahid Sadoughi Hospital, Yazd, Iran with colicky abdominal pain and hematuria. His first presentations were ambiguous genitalia, nausea and vomiting. He was under irregular treatment with prednisolone and fludrocortisone since diagnosis. Parents were relatives. No CAH or other endocrine abnormalities had been diagnosed in his family members.

He was referred because of colicky abdominal pain and hematuria without nausea or vomiting. He weighed 60 kg (height 156 cm; body mass index 24.7 kg/m^2^), blood pressure was 125/80 mmHg, with no orthostatic change. Thyroid and abdomen examination were normal. In breast examination, there was no gynecomastia. Skin examination revealed marked genitalial, areola and gingival hyperpigmentation. Phallus length was 6 cm. testes were not palpable in scrotum. He had axillary and pubic terminal hair (his pubarche was Tanner V). His bone age was 18. Other systemic examinations were normal.

Because of hematuria, some tests were done. In ultrasonography prostate tissue was reported which was smaller than normal size. According to these findings, some other tests were requested which are presented in table I. According to lab findings, treatment started with dexamethasone 0.5 mg/day and fludrocortisone 0.05 mg/day in single dosage and tests were repeated after a month. Karyotyping was performed because of uncertainty in primary diagnosis, which revealed 46XY. For finding the location of testes, ultrasonography and magnetic resonance imaging (MRI) were done and nothing was found in abdomen, inguinal canal or scrotum.

Appendectomy was performed because of persistence of abdominal pain. Despite appendectomy, the abdominal pain continued. Laparoscopy was performed to find the testes which were unrevealing. Inhibin B serum level was measured to find out whether testis tissue was present in the body which was <1 pg/ml and vanishing testis was confirmed. After one month of therapy, serum level of testosterone decreased to level 0.025 ng/ml. Oral consent was obtained from the patient. 

**Table I:** Laboratory data of patient

**Test**	**Result**	**Normal range**
**17-OHP (**ng/mL)	**46 **	0.5-2.4
**Testosterone (**ng/dL**)**	**4.6 **	3-7
**DHEAS (**μg/dL**)**	**3.7 **	199-334
FSH (mlu/mL)	0.1	0.1-3
LH (mlu/mL)	0.1	0.1-3
Serum **ACTH (**pg/mL**)**	**1000 **	10-50
Serum **Cortisol (**μg/dL**)**	**2 **	6-17
Na (mEq/ L)	142	135-145
K (mEq/ L)	4.1	3.5-5.5

17-OHP: 17-Hydroxyprogesterone

DHEAS: dehydroepiandrostenedione sulfate

FSH: Follicle-stimulating hormone

LH: Luteinizing hormone

ACTH: Adrenocorticotropic Hormone

## Discussion

CAH is an autosomal recessive disorder resulting from 21-hydroxylase deficiency (21-OH CAH). More than 95% of cases are caused by 21-hydroxylase deficiency (1). These enzymatic defects in steroid pathway lead to switching the process of aldosterone and cortisol synthesis into other pathways (sex hormones) and over production of intermediate metabolites. Potential clinical effects occur due to these metabolites and other hormones (5). Most patients can have different degrees of symptoms. Lack of aldosterone results in hyponatremia, hyperkalemia, hypotension, acute dehydration and vomiting. Also, it can be life-threatening. Hyperandrogenism is the other problem. It can cause abnormal development in children including abnormal development of genitalia in both male and female (1).

CAH is the most common cause of ambiguous genitalia resulting from hyperandrogenism. This can cause enlarged clitoris, fused labia major and other dysfunction in genital system. Girls are more easily diagnosed but boys have no overt signs except premature pubarche, virilization, hyperpigmentation around the genitalia and penile enlargement (4, 6). It is categorized into two major types. The severe type of CAH is known as classical CAH (salt-wasting) and the milder type is known as non-classical CAH (late onset).The late onset CAH form starts in women at any age. This form presents with normal genitalia at birth in both males and females. 

In fact, this type has similar symptoms to polycystic ovary syndrome. Usually we cannot detect this type in men. In the severe type, loss of salt from the body is the main problem. Hyperandrogenism is much more severe in this type (7). The prevalence of mild type is 1 in 1700 in general population but the prevalence of severe type is 1 in 10000 among Caucasians (8). CAH due to 21-hydroxylase deficiency may affect the final height of these patients (9, 10). Also glucocorticoids can cause growth retardation (11).

We can diagnose CAH in fetus by amniocentesis and chorionic villus sampling. We can treat this to prevent birth defects. After birth, if the child has ambiguous external genitalia, genetic testing and ultrasound can determine the sex (5, 12). Measuring 17 hydroxy progesterone levels is a diagnostic test. Genetic testing can be helpful. Also short ACTH stimulation test can be done (13). In this case, karyotyping was done which the report was 46XY. Lifelong treatment with synthetic glucocorticoid supplementation is necessary. In these patients, replacing hormones such as dexamethasone and fludrocortisones is needed to replace cortisol and aldosterone (14).

The patient also had cryptorchidism. It is the most common congenital abnormality in boys, affecting 2-4% of term infants but its prevalence decreases to about 1% by the age of 1 year. Vanishing testis syndrome (or testicular regression syndrome or anorchia) is a rare birth defect. It may be seen in less than 5% of cryptorchidism cases in which a baby boy is born without testes but the penis and scrotum will form with good development (15, 16). Hormonal and mechanical factors are the most common causes of this condition. Its specific cause is not known, but it thought to be genetic in some cases. It is the result of vascular accident, torsion, or endocrinopathy. The baby would have ambiguous genitalia, which means the child would have parts of both male and female genitals (17). 

The diagnosis of congenital vanishing testes should be suspected in a patient with 46XY karyotype, no detectable testes and lack of secondary sex characteristics. The boy presented in this report, had all these characteristics of the disease (18). Ultrasonography is non-invasive, non-ionizing and very useful technique. It is actually difficult to differentiate between testis and inguinal lymph nodes. MRI is a gold standard diagnostic test (19). In this patient for finding the location of testes, ultrasonography and MRI have been done and nothing was found in abdomen, inguinal canal or scrotum and prostate was normal. Clinical findings are not specifically diagnostic, and must be confirmed by endocrinological evaluation such as GnRH and hCG tests (20). Inhibin B serum level was measured, which was <1 pg/ml, and vanishing testis was confirmed. Also after one month therapy with dexamethasone and fludrocortisone, serum level of testosterone decreased to pre-pubertal level (0.025 ng/ml) which indicates that the origin of testosterone is adrenal and the other tests were not necessary.

In conclusion, because of simultaneous occurrence of CAH and vanishing testes, which is a rare condition, we recommend that karyotyping be done for all patients with CAH.

## Conflict of interest

There is no conflict of interests of each author.
